# Confirmation of unidimensionality of the Dermatology Life Quality Index (DLQI) using a multinational 3,408 patient dataset

**DOI:** 10.1186/s41687-026-01025-0

**Published:** 2026-02-24

**Authors:** Jeffrey Johns, Sam Salek, Faraz Ali, Florence Dalgard, Jörg Kupfer, Andrew Y. Finlay

**Affiliations:** 1 Division of Infection and Immunity, School of Medicine, Cardiff University, Cardiff, CF14 4XN UK; 2 School of Health, Medicine and Life Science, University of Hertfordshire, Hatfield, AL10 9AB UK; 3 National Center for Dual Diagnosis, Innlandet Hospital Trust, Brumundal, Norway; 4 Institute of Medical Psychology, Justus Liebig University, Giessen, Germany

**Keywords:** Dermatology Life Quality Index, DLQI, Validation, Unidimensionality, Item response theory

## Abstract

**Background:**

The Dermatology Life Quality Index (DLQI) was designed to be a simple, practical questionnaire for routine clinical use and is the most widely used tool to measure the burden of skin diseases and assess effectiveness of interventions based on patients’ perspective. The aim of this study was to further validate the DLQI using a multinational European study dataset.

**Methods:**

Data from a cross-sectional study conducted in 13 European countries were analysed. In each dermatology clinic, 250 consecutive adult out-patients were recruited. A wide range of classical test theory and IRT tools were used to investigate unidimensionality and known-group properties.

**Results:**

From 3,635 patients, 3408 completed the DLQI with no missing data. 55.8% of patients were female, mean age 46.6 years (SD = 17.82). The commonest conditions were psoriasis (17.4%), non-melanoma skin cancer (10.8%), infection of the skin (6.7%), hand eczema (6.2%) and acne (6.2%). Using DLQI score banding, the patients’ disease had no effect (*n* = 962, 28.2%), small (912, 26.8%), moderate (674, 19.8%), very large (691, 20.3%) or extremely large effect (169, 5.0%) on their quality of life. Psychometric analyses employing eigenvalues, scree plots, item-response theory (IRT) and very simple structure indicated unidimensionality. Cumulative explained common variance did not significantly increase from 0.618 with additional factors. Confirmatory factor analysis fits for a 1-factor solution were very good and a 1-factor model was optimum. IRT indicated good fit statistics, no misfitting items (infit and outfit), and no local dependencies (LG2 and Q3). Cronbach’s alpha was 0.90. Cohen’s-d effect sizes for DLQI scores between different EQ-5D item levels were moderate (> 0.5) or large (> 0.8), and large between different disease severity levels. Good correlation was found between EQ-5D VAS scale and DLQI score (r^2^ =-0.409, *p* < 0.001). Known group analysis of DLQI score by severity was significant between groups (*p* < 0.001).

**Conclusion:**

Analysis showed good psychometric properties confirming a 1-factor unidimensional model.

**Supplementary Information:**

The online version contains supplementary material available at 10.1186/s41687-026-01025-0.

## Introduction

The Dermatology Life Quality Index (DLQI) was designed to be a practical questionnaire for routine clinical use, with simplicity of reporting and application, a single meaningful summary score, and ease of completion in two minutes [1]. With comparability between studies and over time aided by there being only a single version, the DLQI [2, 3] is the most widely used tool for clinicians and researchers to quantify the impact of skin diseases on patients’ physical and psychosocial functioning as well as to assess the effectiveness of interventions [4]. In a previous systematic review of peer reviewed publications published between 1994 (date of DLQI inception) to 2021, we found 457 studies reporting randomised controlled trials using the DLQI [5] (as primary outcomes in 24) [6]. In another systematic review we found the DLQI used as benchmark for validating > 100 other newly developed patient reported outcome measures [7]. It has also been incorporated in guidelines or registries in > 45 countries [8] and is available in 138 translations [9, 10], However, extensive use of the DLQI does not, by itself, guarantee optimal measurement in all populations and disease severities, and thus further validation and experience in various populations, settings and diseases is always desirable.

Limitations of the DLQI concerning floor and ceiling effects, dimensionality and cultural differential item functioning DIF were highlighted in our previous systematic review [10]. The DLQI has shown good test–retest reliability, internal consistency reliability, responsiveness to change, known group validity, correlation with other patient reported outcomes (PRO)/quality of life (QoL) measures and interpretability or clinical meaningfulness of the scores [10].

Unidimensionality means that a measure has only one construct or dimension (latent trait) and thus one single common factor accounts for all item covariances and all the items predominantly reflect a single underlying construct (latent trait). The unidimensionality of a measure is normally assessed by confirmatory factor analysis (CFA), exploratory factor analysis (EFA), parallel analysis, or item response theory [11]. Unidimensionality implies a single latent factor is hypothesized to account for the correlations among items and is a fundamental requirement of any patient-reported outcome measure if a single score is to be calculated from all the items to provide an overall score based on the construct for that measure. However, serious violations may compromise interpretability of that score, rather than implying a strict all-or-nothing criterion. Given the very wide international use of the DLQI as a single summed score (e.g. in trials, guidelines, and thresholds for clinical decisions), it is important to examine its dimensionality. As described in our recent systematic review on the validation of the DLQI, the current literature gives conflicting messages on its unidimensionality [10].

The aim of this study was to further validate the DLQI using a 13-country European multicentre cross-sectional study dataset with 3,408 patients [12]. This was specifically focused on the dimensionality of the DLQI and item response theory but additionally explored internal consistency reliability and known group analysis.

## Materials and methods

Data were analysed from a European multicentre cross-sectional study [12] conducted in 13 countries (Belgium, Denmark, France, Germany, Hungary, Italy, Netherlands, Norway, Poland, Russia, Spain, Turkey and the UK). Two countries (Norway and Italy) had two centres of collection in different parts of the country, and their data have been combined for the analyses. In each dermatology outpatient clinic, 250 adult out-patients were recruited consecutively. Each questionnaire was in the local language of the country. The dataset contains DLQI raw scores, EQ-5D 3-levels, visual analogue scale and physician assessed disease severity (3-point Likert scale as mild, moderate or severe). Patients with missing DLQI responses were excluded.

Analysis used both classical test theory (CTT) and item response theory (IRT). Construct validity, referring to the degree to which a test measures the theoretical construct it is intended to measure, was evaluated. Dimensionality was investigated by the methods shown in Table 1.

**Table 1:** Comparison table of dimensionality assessment methods with key details (ranked from strongest first to weakest last)

Method	Type	Assumptions	Pros	Cons	Recommended Use	Ref
Parallel Analysis (PA)^1^	Exploratory	Assumes random data comparison is valid	Most accurate for factor retention; widely recommended	Requires simulation; slightly more complex	Primary method for EFA	[13]
Velicer’s MAP^2^	Exploratory	Based on partial correlations	Good empirical performance; complements PA	Less intuitive; needs computation	Use with PA for confirmation	[14]
Confirmatory Factor Analysis (CFA)^3^	Confirmatory	Requires hypothesized model; multivariate normality	Gold standard for testing dimensionality; fit indices available	Needs large sample; model specification critical	Confirmatory phase	[15]
Item Response Theory (IRT)^4^	Confirmatory	Assumes unidimensionality for most models	Item-level precision; strong evidence	Complex; large sample required	After EFA/CFA for item-level checks	[16]
Very Simple Structure (VSS)^5^	Exploratory	Assumes simple structure improves interpretability	Helps decide interpretable factor count	Less common; subjective interpretation	Supplementary to PA/MAP	[17]
Scree Test^6^	Exploratory	Visual elbow indicates factor count	Simple; widely used	Subjective; prone to error	Quick visual check alongside PA	[18]
Kaiser-Guttman Rule^7^	Exploratory	Eigenvalue > 1 indicates factor	Easy to apply	Overestimates factors; outdated	Never use alone; only as heuristic	[19, 20]
Chi-square of Residual Matrix^8^	Exploratory	Tests residual correlations	Formal statistical test	Highly sensitive to sample size; rarely practical	Rarely recommended	[21]

^1^Extracting factors (using EFA) until the eigenvalues of the observed correlation matrix are less than those from random data of the same size (parallel analysis). ^2^Using Wayne Velicer’s Minimum Average Partial (MAP) criterion. ^3^Confirmatory factor analysis. ^4^Item response theory. ^5^Using the Very Simple Structure Criterion (VSS). ^6^Scree Test ^7^Extracting principal components until the eigenvalue < 1 (Kaiser-Guttman rule), plotting and applying the scree test

^8^Extracting factors until the chi-square of the residual matrix is not significant

Using parallel analysis in the R version 4.2.2 (R Foundation for Statistical Computing) Psych package with polychoric correlations, WLS factor method, 100 replications and the quantile of the simulated/resampled data set to the 95th percentile, factors were extracted until the eigenvalues of the real data were less than the corresponding eigenvalues of a random data set of the same size. Due to convergence problems of the algorithm used to compute the polychoric correlations in data with floor effects, in some cases we used smoothing to produce a Gramian polychoric matrix, and random column permutations of the real data matrix (in order to generate the random criterion variables in practice). Additionally, recent work of Garrido et al. [22] states “PA with polychorics is relatively robust to the skewness of the ordinal variables”.

Factor analysis was performed using R with package Lavaan version 0.6–16. Hierarchical clustering analysis was performed using iCluster [23], an algorithm that specifically explores questions about the reliability of the clusters where clusters are formed until either coefficient α Cronbach or β fail to increase. The results were used to guide selection of factors for confirmatory factor analysis (CFA) modelling, along with EFA factor loadings. CFA was performed using R library Lavaan to examine whether the data fit the predetermined 1-factor model [24]. Because the DLQI data were not normally distributed and showed floor-effects, the Weighted Least Squares with Mean and Variance adjusted (WLSMV) estimator was also used, that is designed for ordinal data, is asymptotically distribution free, and does not make distributional assumptions about the observed variables [21, 25].

Very Simple Structure (VSS) [17], an alternative procedure for estimating the optimal number of interpretable factors, was used with promax rotation and factoring method minres (minimum residual) as it is a least-squares approach that does not require normality and is generally more robust for non-normal or ordinal data, minimising residuals without relying on likelihood theory. It applies a goodness of fit test to determine the optimal number of factors to extract; a quasi-confirmatory model, in that it fits the very simple structure (all except the biggest c loadings per item are set to zero where *c* is the level of complexity of the item) of a factor pattern matrix to the original correlation matrix. Wayne Velicer’s Minimum Average Partial (MAP) criterion [14] in R was applied. The MAP criterion computes the average of the partial correlations, and when the average partial correlation stops decreasing and starts increasing with more factors, more individual variability than common variability in the data has been accounted for.

For CFA, fit parameters were used to test whether the proposed 1-factor model was superior to alternative two-factor models. Specifically these 2-factor models were Model1: factor1 items 1–5,7 and factor2 items 6,8–10, Model2: factor1 items 1–7 and factor2 items 8–10 and Model2: factor1 items 1–7 and 10 and factor2 items 8 and 9. Models1 and 3 were based on factorisation from EFA and iCLUST analysis [23] and Model2 factorisation based on iCLUST analysis [23] and Xiao et al. (2018) [26]. Evaluation of model fit was performed using Root Mean Square Error of Approximation (RMSEA), the comparative fit index (CFI), Tucker–Lewis index (TLI), and Standardised Root Mean Square Residual (SRMR) including 90% confidence intervals (CI). The RMSEA expresses the lack of fit per degree of freedom of the model with values interpreted as follows: ≤0.05 = very good; >0.05–0.08 = good; ≥0.10 poor fit. The CFI assesses fit relative to a null model and ranges from 0 to 1 with values of 0.90–0.95 indicating acceptable and > 0.95 good fit. The TLI adjusts for the number of model parameters and is interpreted as for CFI. The Adjusted Goodness of Fit Index (AGFI) is the proportion of variance accounted for by the estimated population covariance and corrects for the number of indicators of each latent variable and should be > 0.95 [27]. The SRMR is the average of the differences between the observed and predicted correlations and has a range from 0 to 1 with values < 0.08 indicating good fit. Although conventional cut-offs (e.g., AGFI ≥ 0.90) are included, we clarify that they serve only as heuristic benchmarks—not definitive rules. As Hu and Bentler argue [28], reliance on multiple indices (e.g., CFI, RMSEA, SRMR) within a multi-index strategy provides a more robust assessment of fit, and strict thresholds should not substitute for substantive judgment but as a heuristic aligned with widely cited guidelines. Kline [29] and others similarly highlight that fit indices must be interpreted in light of sample size, model complexity, theoretical rationale, and overall pattern of results and not dichotomous “pass/fail” interpretation and reflecting current best practices in structural modelling.

High interfactor correlations (> 0.78) suggested 2-factor solutions were not distinct, supporting unidimensionality. However, substantive interpretation of the two-factor content and formal tests (Satorra-scaled Δχ² tests [30, 31], robust fit index and BIC comparison) were performed to rule out theoretically meaningful multidimensionality. When data are non‑normal, the usual model‑fit chi‑square (from normal‑theory ML) is often inflated. The SB correction rescales that chi‑square using a scaling factor. As our models used ordered = TRUE and estimator = “WLSMV” the SB method defaulted to a Satorra (2000) style scaled/shifted difference test. Robust fit indices (CFI/TLI/RMSEA) in Lavaan also use these scaled statistics. The robust difference test uses a function of these standard statistics, so the p-value is valid for the Satorra–Bentler method. Fornell–Larcker discriminant validity [32] using factor covariance r² vs average variance extracted was tested, and HTMT (Heterotrait–Monotrait ratio), a modern and more sensitive method for assessing discriminant validity in CFA assessed [33, 34].

Reliability was estimated using Cronbach’s alpha [35, 36] and several omega coefficients [37] (Revelle’s omega hierarchical [38], Bentler’s omega total [39, 40] and McDonald’s omega total [41]) were calculated with semTools in R. Cronbach’s alpha assumes tau-equivalence and is sensitive to item number and redundancy, may underestimate reliability when loadings differ. Omega coefficients are factor-analytic reliability estimates that allow congeneric items. Bentler’s omega and McDonald’s omega both estimate the reliability for total scores i.e. proportion of variance attributable to all common factors (Bentler’s is SEM-based model-implied covariance, while McDonald’s is often implemented using observed covariance, although this distinction is implementation-specific, not theoretical), whereas Revelle’s hierarchical omega (ωₕ), derived via a Schmid–Leiman transformation, estimates reliability attributable to a general factor only. Omega estimates are generally preferred to alpha when factor loadings are unequal and the measurement model is well supported. Values ≥ 0.70 are often interpreted as acceptable reliability (context-dependent e.g. exploratory vs. clinical use), though multidimensional scales may exhibit lower hierarchical omega values. Internal consistency was also determined by Cronbach’s alpha with item deletion [36, 42] calculated with IBM SPSS Statistics version 27, and correlations and effect sizes were all calculated in R.

Item response theory (IRT) was performed by R or IRTPRO version 6.0 09.22 (Scientific Software International Inc., Chapel Hill, NC, USA), using Rasch [43] and Graded Response Models (GRM) [44] with 4 categories. Several key assumptions underlie the IRT framework, including (i) unidimensionality of the measured trait, (ii) local independence, (iii) monotonicity, and (iv) item invariance [44]. Unidimensionality assumes that a set of items on a scale measure just one thing in common. Local independence means that every item on a PRO measure is statistically independent of responses to all other items on the measure, conditional upon the latent trait. Local dependence (LD) exists when there is additional systematic covariance among the items. It can occur when pairs of items have highly similar content or between sequentially presented items in a test. LD was assessed by LDG2 [45] and Q3 [46] in R. Items with LDG2 > 10 or Q3 residual correlations > 0.20 were flagged for potential local dependence consistent with established guidelines of Chen & Thissen [45] and Yen [46]. Items showing local dependence need to be reviewed for content overlap for consideration to be retained or removed based on theoretical relevance and impact on model fit.

Monotonicity refers to the phenomenon in which the probability of endorsing an item will continuously increase as an individual’s trait level increases e.g. the probability of selecting a response category indicative of better health should increase as underlying health increases. Item invariance is a property of IRT that states that item parameters are constant even if estimated from different samples, though empirical checking is required to substantiate robustness of IRT results. This means that the characteristics of the item parameters and latent trait are independent of the sample characteristics within a population.

## Results

**Fig. 1:**
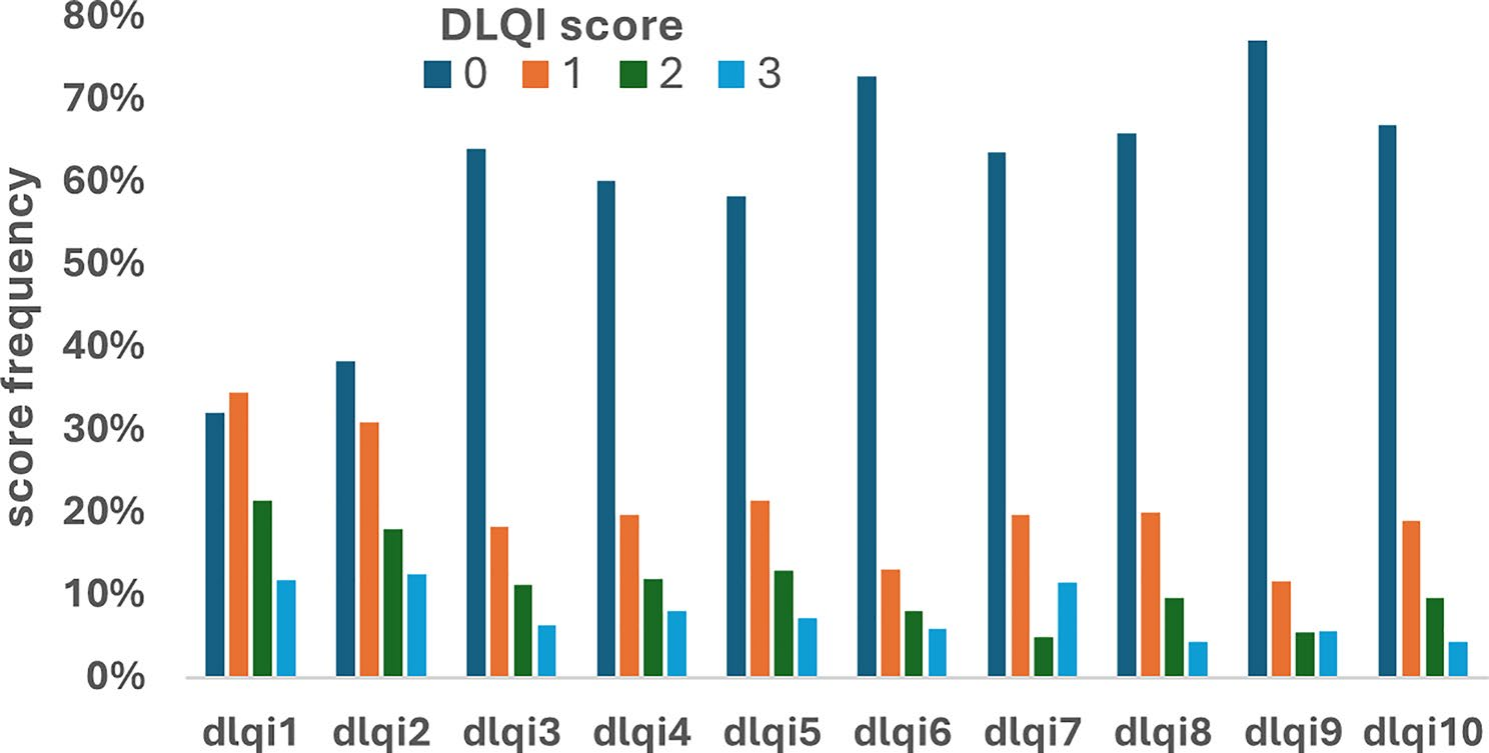
Individual item score distributions for the ten DLQI items

From 3,635 patients, 3,408 patients completed the DLQI questionnaire with no missing data and were used in the analysis: this was the dataset used in the current study. The 227 (6.3%) who did not complete the DLQI did not differ from completers based on key demographics. Of the 3,408 patients, 3,350 patients completed the EQ-5D questions, and 3,250 the EQ-5D VAS Eq. 5D-VAS. 55.8% of patients were female and mean age was 46.6 years (SD = 17.8). The mean DLQI sum score was 5.03, mean EQ-5D VAS score 75.35. The DLQI score distributions are shown in Fig. 1. A strong Spearman’s correlation was seen between DLQI scores and EQ-5D VAS (r^2^=-0.409, *p* < 0.001) and the average DLQI score and the average EQ-5D VAS score for each of 27 diseases (r^2^=-0.609, *p* < 0.001) (Figure S1). The distribution of EQ-5D VAS scores is shown in Figure S2.

The commonest conditions reported were: psoriasis (17.4%), non-melanoma skin cancer (10.8%), infection of the skin (6.7%), hand eczema (6.2%), acne (6.2%), nevi (5.0%), atopic dermatitis (4.5%), benign skin tumors (4.2%), eczema (contact dermatitis) (4.1%) and venous insufficiency/leg and other ulcers (2.8%). Plots of average DLQI and EQ-5D VAS score versus disease are given in Figures S3 and S4 respectively. Physician reported disease severity of patients was recorded in only 2,902 patients (85.2%), as mild 379 patients (11.1%), moderate 1371 (40.2%) and severe 506 (14.8%). Disease severity was not reported for 379 (11.1%) of the total 3,408 participants included in the study. Average DLQI scores versus physician assessed disease severity is plotted in in Figure S5.

Applying the DLQI score meaning banding [47], the patient’s disease had no effect (DLQI score 0–1, *n* = 962, 28.2%), small effect (DLQI score 2–5, 912, 26.8%), moderate effect (DLQI score 6–10, 674, 19.8%), very large effect (DLQI score 11–20, 691, 20.3%) or extremely large effect (DLQI score 21–30, 169, 5.0%) on their QoL. This means that 860 (25.2%) of patients experienced a very large impact on their QoL (DLQI score > 10), a level where alarm bells should start ringing as further action would be required to support them.

Inter-item correlation values were between 0.15 and 0.65 indicating good correlation, with no values < 0.15 (indicating poor correlation) (Supplementary Appendix Table S1). No item had a value higher than 0.65 (items correlated to a greater extent and may be repetitive in measuring the intended construct). Mean inter-item-correlation was 0.472. Polychoric correlations of DLQI items are shown in Table S2.

**Fig. 2:**
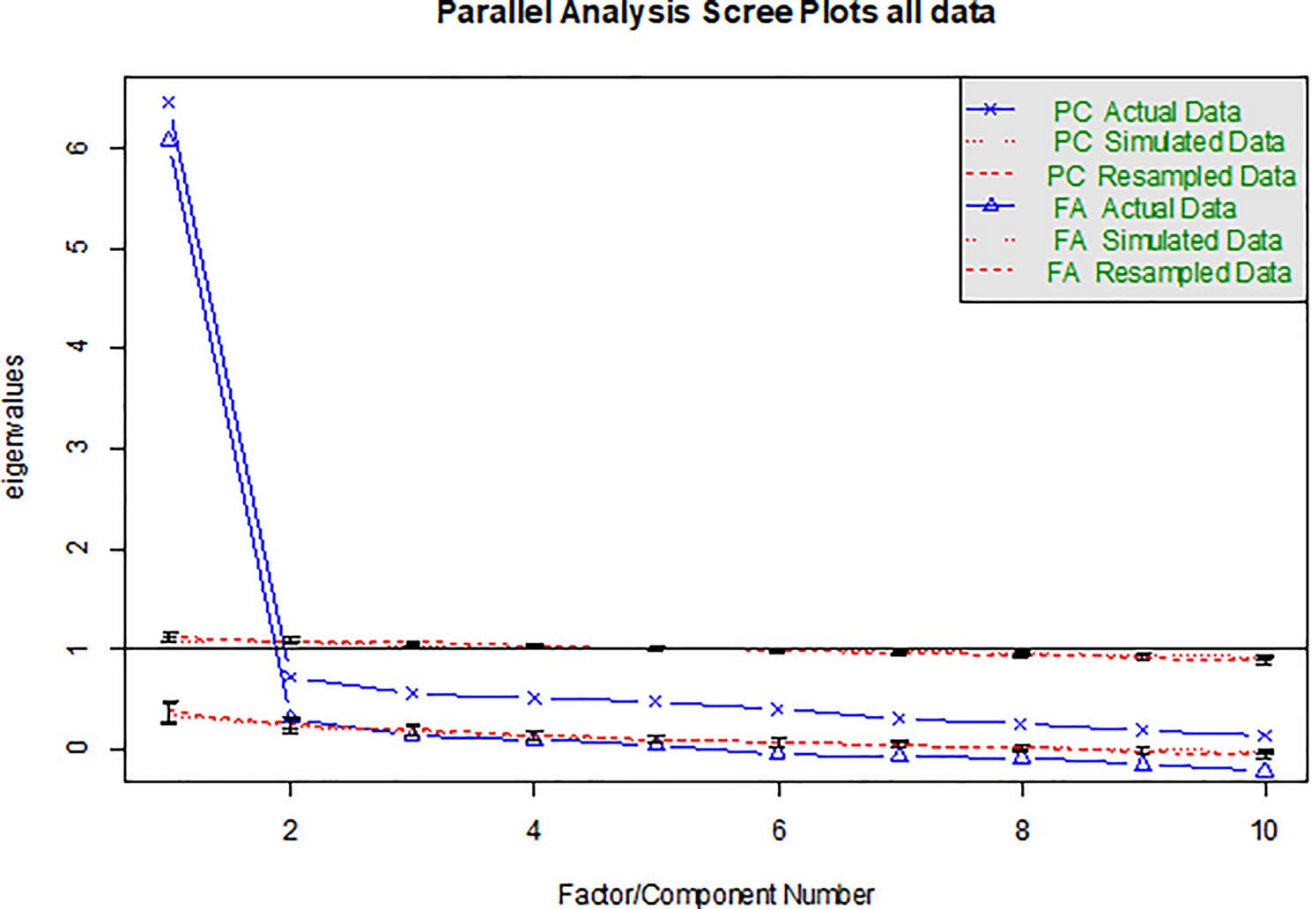
Parallel analysis scree plots

A Kaiser-Meyer-Olkin (KMO) factor of 0.92 and Bartlett sphericity test (χ^2^ = 16119, *p* < 0.001) indicated that the variables were correlated and gave support for factor analysis. The chi-square of the residual matrix from factor analysis was significant after extracting only 1 factor, indicating the model does not perfectly reproduce the observed correlations (which is expected in large samples) (χ^2^ = 1300, *p* < 0.0001 for the residual matrix). Extracting principal components gave eigenvalues of 4.79, 0.31, 0.14, 0.09 and 0.05 and a scree plot (not shown) indicating a 1-factor solution. A parallel analysis scree plot (Fig. 2) shows a distinct change of slope after 1 factor. VSS complexity 1 [17] using promax rotation achieved a maximum of 0.9, which is very high, suggesting a single factor structure. BIC was a maximum of 1042 with 1 factor (Figure S6) indicating model selection criteria favouring the simplest solution. Adding more factors reduced fit progressively and this pattern is typical when the data are essentially unidimensional. Wayne Velicer’s MAP criterion [14] gave the optimal number of components to extract as one (MAP = 0.023 for 1 factor, rising to 0.036 for 2 and 0.057 for 3). The factor fit of the complete model computed as the proportion of the total squared correlations in the observed correlation matrix that is reproduced by the full factor model (with all estimated loadings, not simplified) was 0.90 for 1 factor, decreasing to 0.75 for 2 and 0.066 for 3, on a 0–1 scale, where higher means better reproduction of the observed correlation matrix.

The EFA for a 2-factor solution gave high cross-loading on item 8 (0.39 and 0.51), i.e. loading greater than the maximum recommended of 0.32 on each factor (0.32^2^ ≈ 0.10, which equates to approximately 10% overlapping variance with the other items in that factor) [48]. Additionally, the accepted commonly used operational rule supported by simulation evidence [49, 50] that primary loading should exceed secondary loading by at least 0.20 is also violated (difference = 0.12), further indicating item 8 does not clearly belong to either factor, making interpretation problematic.

The CFA for a unidimensional, and two 2-factor solutions are shown in Table 2. Loading on items only minimally increased ( < = 0.05 change, except for item 8) from 1 to 2-factor solutions, and cumulative explained common variance only minimally increased from 0.618 for 1-factor with addition of further factors. The CFA fit statistics for the 1-factor solution were good.

**Table 2:**
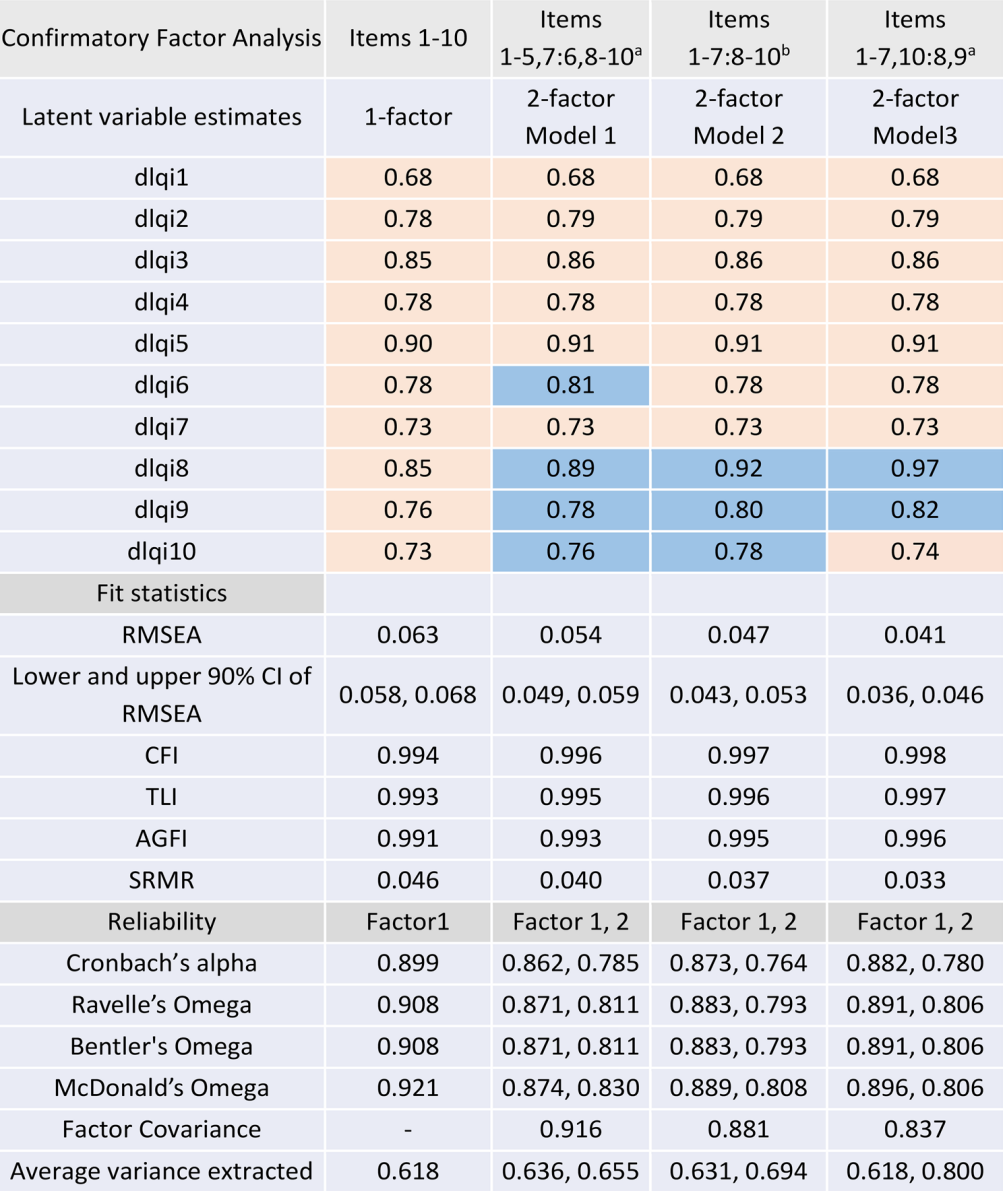
Confirmatory factor analysis

Blue colour indicates second factor

(a) Factorisation based on EFA and iCLUST analysis [23] (b) Factorisation based on iCLUST analysis [23] and Xiao et al. (2018) [26]

Values for RMSEA and SRMR < 0.08 indicate acceptable goodness-of-fit [51]

Values of CFI > 0.96, TLI > 0.95 and AGFI > 0.90, indicate good fit [21, 27]

Alpha: Coefficient alpha uses the item covariance (or correlation) matrix [36]

Ravelle’s Omega: Cutoff > 0.7. Coefficient omega is a measure of composite reliability computed using the item factor loadings and uniqueness from a factor analysis i.e. total variance is the explained variance from the factor of interest plus residual error plus residual covariance [52]

Bentler’s Omega: Uses total implied variance from all factors (full implied covariance matrix) i.e. reliability of a measure without controlling for another factor [52]

McDonald’s Hierarchical Omega: This formula is the most conservative method in calculating coefficient omega and uses observed covariance matrix instead of model-implied covariance matrix to calculate the observed total variance. If the model fits the data well, the third coefficient omega will be similar to the other two [41, 52]

Average variance extracted is calculated from polychoric (polyserial) not Pearson correlations

Global fit statistics are all excellent and very close: 1-factor: RMSEA = 0.063; CFI = 0.994; TLI = 0.993; SRMR = 0.046 and best 2-factor (Model3): RMSEA = 0.041 (CI 0.036–0.046); CFI = 0.998; TLI = 0.997; SRMR = 0.033.

Fit indices change over the 1-factor model are shown in Table 3. ΔCFI ≤ 0.004 and ΔTLI ≤ 0.004 for all models, and by common heuristics (ΔCFI ≤ 0.01; ΔRMSEA ≤ 0.015), these are marginal changes in incremental fit. Model 3’s RMSEA gain (− 0.022) is the largest and exceeds the 0.015 “small change” rule of thumb, but the incremental indices changed very little [28, 53, 54]. SRMR steadily improves, but changes are small (0.013 at most). ΔRMSEA changes by 0.009, 0.016, and 0.022 respectively across the 2-factor models.

**Table 3:** Fit indices change over the 1-factor model (deltas)

Model (factor split)	ΔCFI	ΔTLI	ΔRMSEA	ΔSRMR
2-Factor Model 1 (1–5,7: 6,8–10)	+ 0.002	+ 0.002	−0.009	−0.006
2-Factor Model 2 (1–7: 8–10)	+ 0.003	+ 0.003	−0.016	−0.009
2-Factor Model 3 (1–7,10: 8,9)	+ 0.004	+ 0.004	−0.022	− 0.013

Factor correlations for 2-factor models show discriminant validity concern with very high factor correlations: 0.916, 0.881, 0.837. These are immediately around or above the 0.85–0.90 range often cited as too high for distinct factors (poor discriminant validity). Convergent validity (AVE) is good (≥ 0.50) for all factors (≥ 0.618), with factor2 in the 2-factor model3 very high (AVE = 0.800).

With Fornell–Larcker discriminant validity [32], (using factor covariance r² vs. average variance extracted, AVE) for 2-factor Model 1: *r* = 0.916 so r² =0.839 with both AVEs (0.636, 0.655) < 0.839 fails, for Model 2: r² =0.776 with both AVEs (0.631, 0.694) < 0 0.776 fails and for Model 3: r² =0.700 with AVE1 = 0.618 < 0.700 fails and AVE2 = 0.800 > 0.700 passes but is partial at best, indicating limited discriminant validity across the 2-factor solutions. HTMT point estimates of 0.892, 0.863 and 0.863 for CFA 2-factor models1, 2, and 3 respectively showed that the constructs are highly correlated and discriminant validity is marginal under lenient criteria (HTMT < 0.90) but not supported under recommended strict criteria (HTMT < 0.85) [33, 34].

Loadings are high across models for all items (≈ 0 0.68–0.97). Items 8–9 are very strong in the 2-factor solutions (up to 0.97), which may reflect a content cluster (or local dependence) rather than a truly distinct factor.

2-factor model3 places items 8–9 together (Factor 2) and moves item 10 to Factor 1 (so Factor 2 has only 2 items). Two-indicator factors are identifiable but generally not ideal for stable latent measurement unless there is strong theory and very high loadings.

Robust fit indices and the Satorra-scaled Δχ² tests and increasing BIC all gave statistically better fits for the three 2-factor models than the 1-factor model. When several competing CFA models all beat a single-factor baseline on robust fit indices and the Satorra-scaled Δχ² test, the choice should be driven by a structured decision framework that balances statistical evidence, parsimony, and substantive theory. Statistical significance alone does not guarantee substantive validity. As none of the two-factor models substantially improve fit statistics, average variance extracted, factor loadings or reliability, and show large factor covariance, decreased parsimony and do not support the design construct, a single factor should be accepted.

Cronbach’s alpha [36] was 0.90 (Table 2) and did not increase if any item was deleted (Table S3), indicating that no items should be removed because the higher alpha for 10-items indicates greater reliability. Omega values were > = 0.9 for the 1-factor model, the third coefficient omega was similar to the other two (indicating good model fit), but omega factors were much lower for 2-factor solutions.

### Item Response Theory

**Fig. 3:**
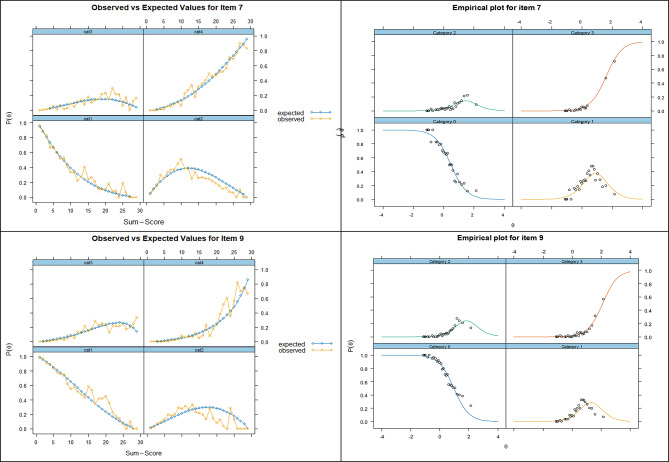
Observed versus expected values and empirical plots for DLQI items 7 and 9

All items show monotonicity i.e. as the trait level increased, the probability of a correct response also increased. Fit statistics for the graded response model (GRM) were RMSEA of 0.04, SRMSR 0.055, TLI 0.936, CFI 0.962. All items gave a signed chi-squared test [55] of < 0.05 and all items had infit and outfit values between 0.5 and 1.5 and are therefore considered to be productive for measurement (Table S4) [56]. Two items (7 and 9) showed disordered thresholds, and for 1 level only (Figure S7). This is a recognised issue [10], but disordered thresholds do not imply multidimensionality by themselves. A scale can be perfectly unidimensional but still have poorly functioning categories. Threshold disordering alone is not evidence of violating unidimensionality: [57] as stated by Adams et al. “Disordered thresholds often indicate problems with response category semantics rather than a violation of unidimensionality” [58]. Disordered items are noted here, but are outside the scope of this study. Test information and standard errors for the DLQI response are shown in Figure S8. However, all 10 items showed good fit for observed vs. expected values and empirical plots including 7 (Over the last week, has your skin prevented you from working or studying?) and 9 (Over the last week, how much has your skin caused any sexual difficulties?), which often lack spontaneous response, accounting for differences in question difficulty (observed vs. expected values and empirical plots for all items shown in Figure S9). However, Fig. 3 shows that from the observed versus expected values and empirical plots for these two items there is excellent fit to the data, but with a slight lack of data for one response level. Observed versus expected values and empirical plots for all items are shown in Figure S9.

No violation of local independence was seen as there were no residual correlations among the items after controlling for the dominant factor for LDG2 [45]; all standardised values were < |0.2| with *p* < 0.001 (Table S5), the COSMIN accepted cutoff [56]. Mean and standard deviation of LDG2 were 0.036 and 0.079, below the accepted cutoff of 0.05 and 0.2 respectively. In addition, all Yen’s Q3 [46] weighted statistics were below the COSMIN accepted cutoff of |0.37| and all *p* < 0.001 with mean=-0.093 and SD = 0.108 (Table S6) [56]. Pearson’s estimate for goodness-of-fit gave χ^2^ = 4,953,751, *p* < 0.0001.

Item invariance was shown by comparing the item parameters of several subsets of the data (by country). Items parameters were similar for each country IRT calibration and there was no difference in the ordering of item location point on the theta (difficulty) scale for each of the data subsets modelled. This gives good evidence that the DLQI items are interpreted similarly by the different samples (data subsets).

When using an anchor measure (e.g., a global rating or external criterion) to validate a PRO or QoL measure in known-groups analysis, the correlation should be at least ≥ 0.30, which is often considered the minimum for meaningful association (especially if constructs are related but not identical) [59, 60] and Cohen’s [61] rules of thumb recommends 0.30–0.35 as a correlation threshold to define an acceptable association between an anchor and a PRO change score. Spearman’s rho intervariable correlations with DLQI sum score for EQ-5D were: EQ-5D mobility 0.211, EQ-5D self-care 0.257, EQ-5D usual activities 0.370, EQ-5D pain/discomfort 0.409, EQ-5D anxiety/depression 0.365, EQ-5D VAS, -0.409, for age − 0.119 and disease severity (physician assessed) 0.407 (all *p* < 0.001). All of these translate to r correlations > 0.45, well above the threshold as acceptable anchors. Although DLQI and EQ-5D capture related but distinct aspects of health-related QoL, mapping of DLQI scores to EQ-5D utility scores has been well established [62, 63].

Cohen’s d effect sizes [61] for total DLQI score between different EQ-5D item levels were moderate (> 0.5) or large (> 0.8), and between disease severity levels were large (Table 4).

**Table 4:**
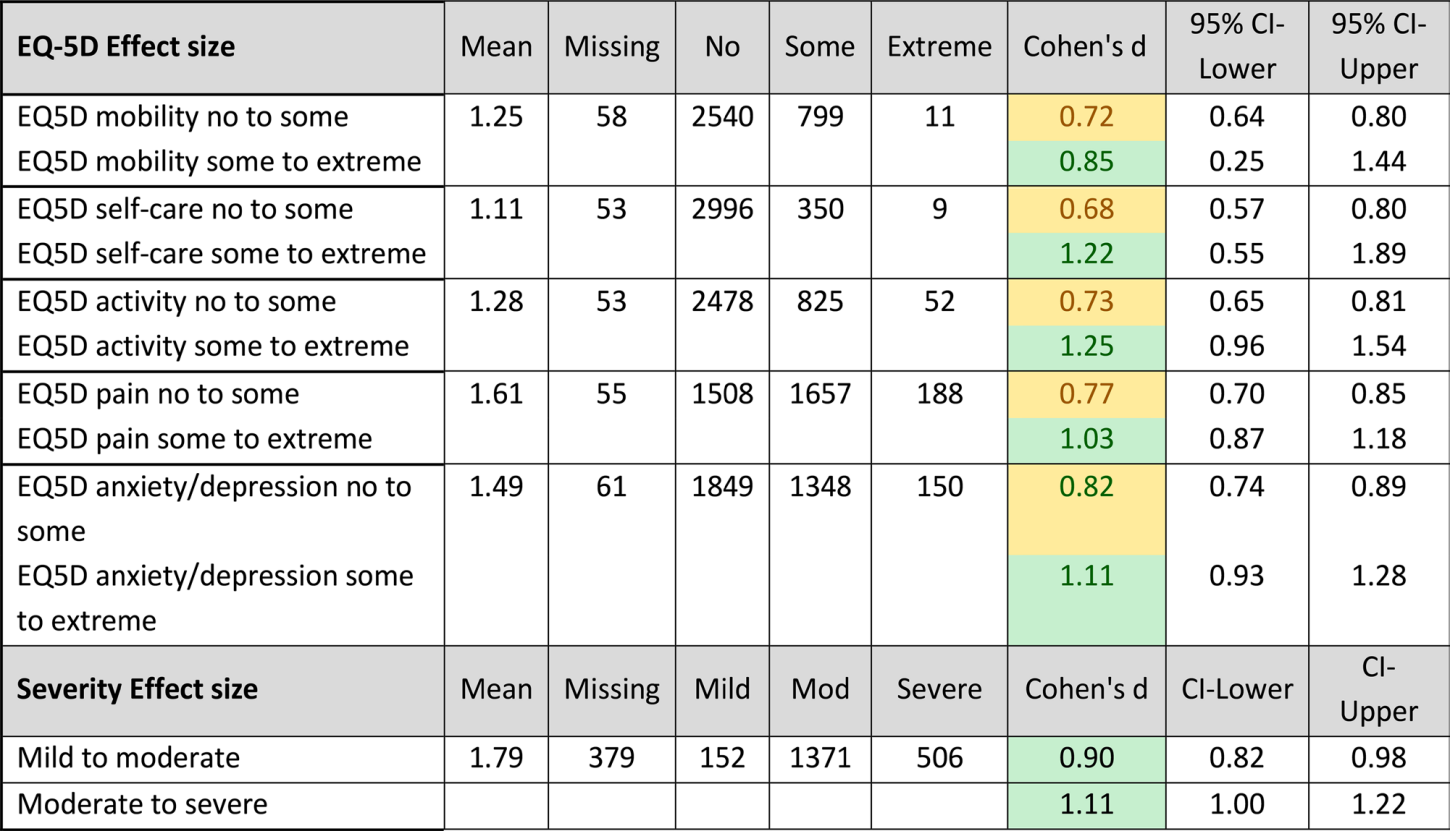
Cohen’s d effects sizes for DLQI sum scores between EQ-5D levels and between physician assessed disease severity levels

Effect sizes are as follows: 0.2 = small effect, > 0.5 = moderate effect (yellow), > 0.8 = large effect (green)

Known group validity analysis of DLQI total score by severity was significant between groups (Kruskal-Wallis Test *p* < 0.001) (Fig S10).

## Discussion

The results of this study showed, through many analytical methods including factor extraction, eigenvalues, parallel analysis. VSS, Wayne Velicer’s Minimum Average Partial (MAP) criterion, CFA and IRT, that a single factor is sufficient to describe the construct of the DLQI, as it was originally designed. The CFA fit variables CFI, TLI and AGFI were all > 0.991 and SRMR was 0.046 indicating an excellent fit to the 1-factor model, while the RMSEA of 0.063 was good (0.05–0.08). The two-factor CFA solutions investigated also had very high covariance between factors (0.89, 0.84, 0.78) indicating that these factors were very highly correlated and should be considered as a single factor. While high interfactor correlations (> 0.78) do not by themselves prove unidimensionality, they show the two latent variables are strongly associated, but they can still capture distinct substantive content (e.g., symptoms vs. psychosocial impact).

As the primary use of the DLQI is to produce a single DLQI severity score, retaining the 1‑factor model as the best overall representation based on CFA is completely defensible:

1. Practical equivalence in incremental fit: ΔCFI/ΔTLI ≤ 0.004 across 2-factor variants—negligible changes by common thresholds [53, 54].
2. High factor correlations and Fornell–Larcker failures [32] and HTMT point estimates below criteria indicate weak discriminant validity of the 2-factor structures, pointing to near‑unidimensionality.
3. Parsimony: With essentially equivalent fit on CFI/TLI/SRMR and strong loadings on one factor, the simpler one-factor model is preferred [21, 28, 29].

Additionally, parallel analysis suggested one factor for the DLQI, consistent with unidimensionality, however this is not definitive evidence by itself. Unidimensionality requires checking whether all items measure a single latent trait adequately which is why CFA, eigenvalues, VSS, MAP, reliability and scalability indices (e.g., Cronbach’s α, McDonald’s ω, etc.) and IRT were also performed. More recent work of Garrido et al. [22] states “PA with polychorics is relatively robust to the skewness of the ordinal variables. In light of these findings, we recommend the use of PA with polychoric correlations”. Additionally we used smoothing to give non-Gramian polychoric matrices if necessary and random column permutations of the real data matrix in order to generate the random criterion variables in practice) for dimensionality assessment of the ordinal-level data. Furthermore, the maximum levels of accuracy were generally achieved in the unidimensional condition i.e. for one factor.

A χ² of 1300 with *p* < 0.0001 for the residual matrix means the one-factor model does not fit the data under the exact-fit test—the residual correlations left after extracting one factor are too large to be attributed to sampling error. In other words, the items may not be adequately unidimensional, or there are additional sources of covariance (extra factors, cross-loadings, or correlated errors) that a single factor fails to capture. A significant χ² rejects that hypothesis, indicating misfit. Because χ² is highly sensitive to sample size, it often turns significant in large samples even when misfit is modest [21].

We recently published a systematic review that compiled data from 207 peer reviewed studies describing validation aspects of the DLQI on 58,828 patients across 49 different countries on the validation of the DLQI [10]. This review identified 28 studies where factor analysis or item response theory was used to examine the dimensionality of the DLQI. These reached different conclusions, identifying from one to four factors. A recommended 20:1 subject to item ratio gives *n =* 200 for the 10-item DLQI, but may still give error rates well above alpha = 0.05 level [50]. Generally, the studies with few data (*n* < 200, 14 out of 28 studies) did not find unidimensionality. Three analyses [64–66] with *n* > 200 (*n* = 900, 1286 and 425) supported the unidimensionality of the DLQI. Exceptions with *n* > 200 were Qi et al. who found a two-factor solution with EFA using SPSS accounting for 61.45% of the variance for a Chinese alopecia group (*n* = 698), with items 2 and 9 in the second factor. No fit statistics were given, and no CFA was performed. Xiao et al. [26] investigating a Chinese population with lifetime arsenic exposure (*n* = 465) also identified two factors with EFA but had models with poor fit statistics and no CFA was performed. Nijsten et al. [67] who studied 450 psoriasis patients from five European and one US centre reported that the DLQI and Skindex-29 scales both significantly misfit the Rasch model in part owing to individual item misfit and threshold disorder. The DLQI showed significant individual misfit (items 1, 3, 5, 8, and 10), three items had reversed thresholds, and (mean item interaction 0.00, SD 0.45; mean person interaction − 1.29, SD 1.28; item–trait interaction, *P* < 0.0001). However, no model fit statistics were provided and no non-Rasch models e.g. PCM or GRM were explored, and analysis based on country data had too small datasets (*n* < 75) to give reliable results. In summary, many previous studies investigating the dimensionality of the DLQI used small datasets, and there is a lack of methodological reporting of handling of non-normal data and floor effects, reporting fit statistics, and choice of models (CFA or non-Rasch models). Methodological heterogeneity (e.g. disease populations, cultures) and DLQI’s documented issues such as floor effects/DIF need to be carefully weighed against the strength of this large-sample study.

IRT of this DLQI dataset indicated good fit statistics, and all items had infit and outfit values between 0.5 and 1.5. Observed versus expected values and empirical plots showed good data fit. All items show monotonicity, item invariance and no violation of local independence was seen. Although two items showed disordered thresholds (for 1 level only), it has been shown that response categories differentiate between participants with different trait levels despite reversed thresholds and that category disordering can be analysed independently of the ordering of the thresholds [68]. Reversed thresholds are often merely a consequence of low frequencies in the response categories concerned and are unlikely to affect the order of the rating scale [58, 69]. Furthermore reversed thresholds often only occur in subgroups of participants and thus researchers should think more carefully about collapsing response categories due to reversed thresholds [68].

Known-group analysis is a type of construct validity that measures an instrument’s ability to detect hypothesized differences among distinct (independent) groups. Groups are generally defined using another independent measure [70], for example DLQI score bands of Hongbo et al. [47] Group differences are then determined using a statistical test. The effect size can also be determined [71]. The DLQI also showed good known group discrimination with Cohen’s-d effect sizes moderate or large using EQ-5D items as anchors, or large with physician assessed disease severity as an anchor. Known group validity analysis of DLQI total score by severity was significant between groups, however, this result must be interpreted with caution, as we were unable to perform inter rater reliability between the physicians conducting these assessments of severity and this may introduce bias and incompleteness.

Known limitations of the DLQI, such as reduced sensitivity in milder disease or concerns about some item content (e.g. sexual life, work/sport items) in particular cultures have been previously noted. Despite its widespread use and good overall psychometric performance, some studies report floor effects, item misfit, or cultural challenges. However, the analysis of the data presented in this study confirm that these issues have limited effect on the unidimensionality of the DLQI scale.

A limitation of this study was the low overall DLQI scores coupled with pronounced floor effects, restricting score variability and attenuating inter-item correlations, which can reduce statistical power and mask multidimensionality. This range restriction reduces the sensitivity of dimensionality assessments, often masking secondary factors [72, 73] that emerge at higher severity levels. Factor analysis and IRT modelling rely on adequate variance and category usage; when most respondents endorse only the lowest response options, polychoric correlations [74] and item thresholds can become unstable. Consequently, multidimensionality or violations of monotonicity and invariant item ordering may remain undetected in mild samples. In parametric IRT (e.g., Rasch models), person–item targeting gaps may leave higher-severity items untested, concealing multidimensional features [75]. Similarly, in nonparametric IRT frameworks such as Mokken scaling, low variance and empty higher categories depress scalability coefficients (H, Hi) and impair detection of monotonicity violations or invariant item ordering [76]. Further investigation of item and category function, scalability and monotonicity using Mokken analysis needs to be performed.

Using a large, pooled, diverse dataset across 13 countries increases statistical power and enhances generalizability, but it simultaneously introduces potential threats to measurement validity. Cross-national pooling can obscure culture-specific item functioning, linguistic nuances, and response styles that differ across countries. Certain items of the DLQI might perform differently across cultures and severity ranges which could limit comparability or responsiveness. Adhering to the principle of cross-cultural adaptation should minimise these effects. However, even when instruments are translated using rigorous methods, subtle cultural connotations may lead to DIF, non-invariant factor loadings, or shifts in thresholds. Further investigation of these issues using country-specific CFAs or IRT models, multi-group invariance testing (configural, metric, scalar), DIF analyses by country or language group and examination of culturally patterned response biases is needed [77], but beyond the scope of the present study.

The results supporting the unidimensionality of the DLQI are very encouraging, considering that the dataset comes from pooling of data collected from 13 Europe countries and many different sites, and representing 13 different language versions of the DLQI. The construct validity of the DLQI would seem to be retained across the translations, although further analysis is required to confirm this. Additionally, previously reported [65, 67] DIF and evidence of good longitudinal response of the DLQI (ability to detect change over time) needs to be investigated further.

## Conclusion

The DLQI showed good psychometric properties in this large study dataset using both classical test theory as well as IRT, supporting unidimensionality of the DLQI.

## Supplementary Information

Below is the link to the electronic supplementary material.

Supplementary Material 1

## Data Availability

Data is available on reasonable request.
