# Supplementary material for: Confirmation of unidimensionality of the Dermatology Life Quality Index (DLQI) using a multinational 3,408 patient dataset
Source: J Patient Rep Outcomes. 2026 Feb 24;10:49. doi: 10.1186/s41687-026-01025-0 (PMC13038833; doi:10.1186/s41687-026-01025-0)

Supplementary Appendix

Table of Contents

1. Figure S1. Average DLQI score versus average EQ-5D VAS score for each of 27 diseases
2. Figure S2. Distribution of EQ-5D VAS
3. Figure S3. Average DLQI scores for each disease (bars indicate SD, bar number is number of patients)
4. Figure S4. Average EQ-5D VAS scores for each disease (bars indicate SD, bar number is number of patients).
5. Figure S5. Average DLQI scores versus physician assessed disease severity
6. Table S1. Correlation matrix between DLQI items
7. Table S2. Polychoric correlations of DLQI items
8. Figure S6. Very Simple Structure plot using VSS varimax rotation
9. Table S3. Internal consistency reliability* of the DLQI
10. Table S4. Infit and outfit statistics (Rasch model)
11. Figure S7. Item response curves for the DLQI
12. Figure S8. Test information (blue) and standard errors (red) for the DLQI response
13. Figure S9. Observed versus expected values and empirical plots for DLQI items
14. Table S5. Local dependence LD2
15. Table S6. Q3 statistics. All Q3 < 0.37
16. Figure S10. Known group validity analysis of DLQI total score by physician reported disease severity

Figure S1. Average DLQI score versus average EQ-5D VAS score for each of 27 skin diseases

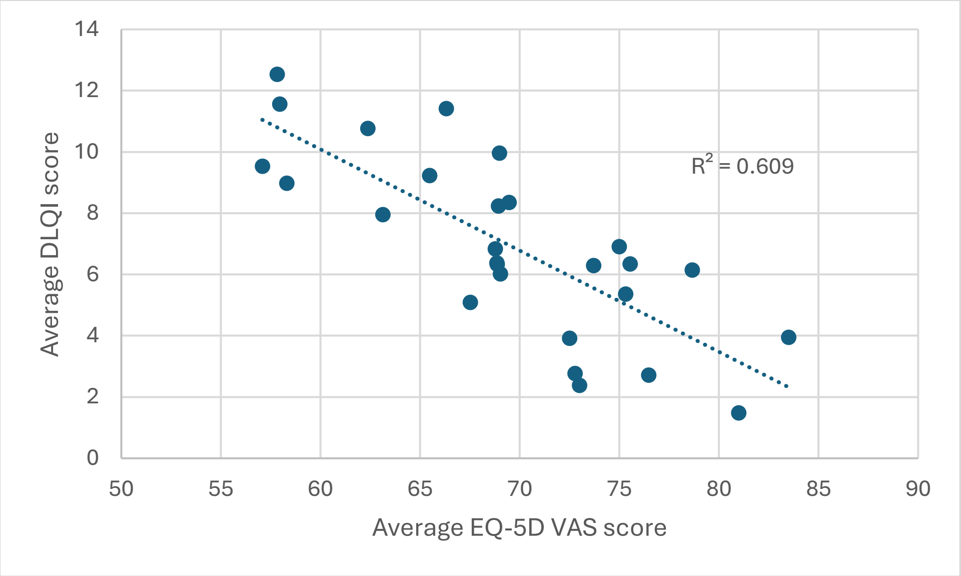

Figure S2. Distribution of EQ-5D VAS

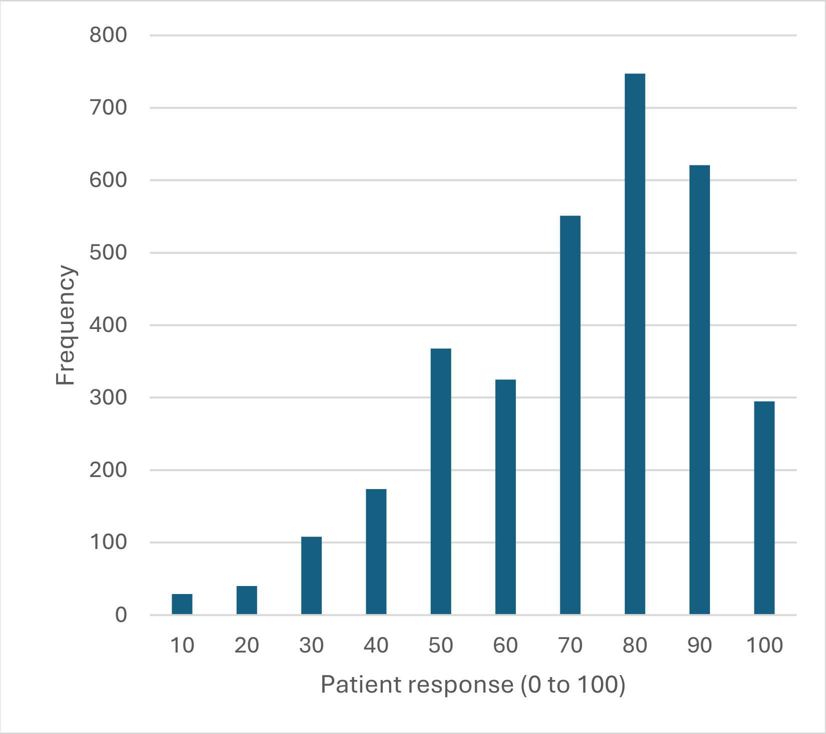

Figure S3. Average DLQI scores for each disease (extended lines indicate SD, bar number is number of patients)

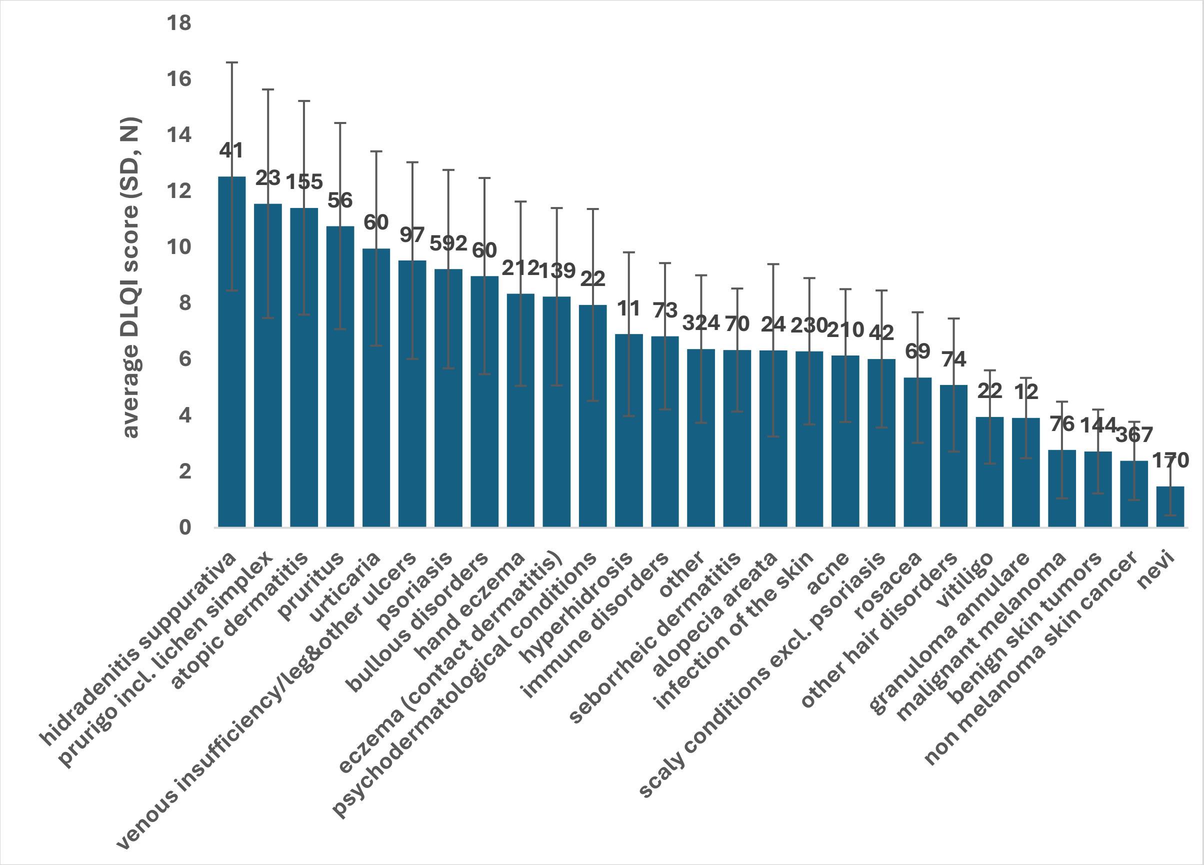

Figure S4. Average EQ-5D VAS scores for each disease (extended lines indicate SD, bar number is number of patients)

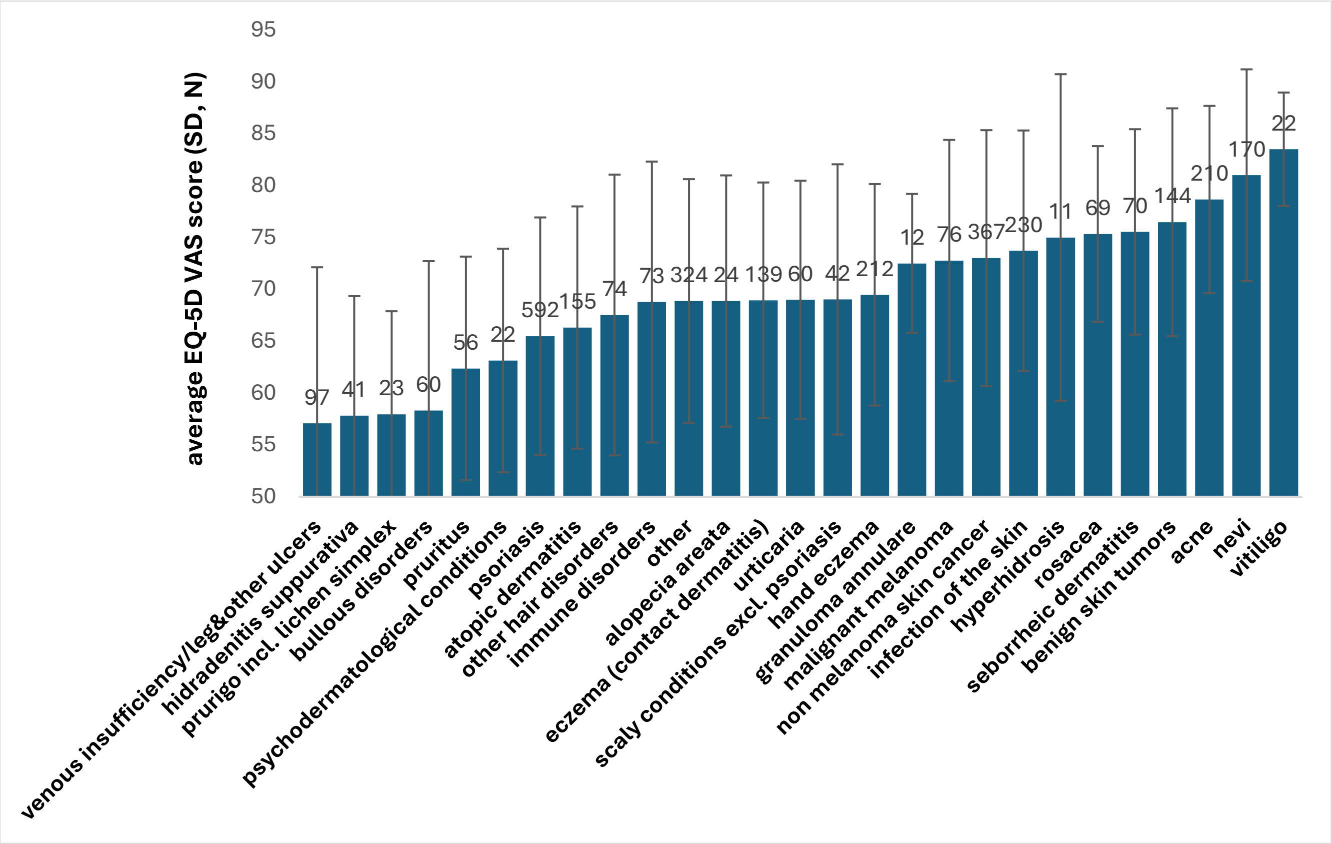

Figure S5. Average DLQI scores versus physician assessed disease severity

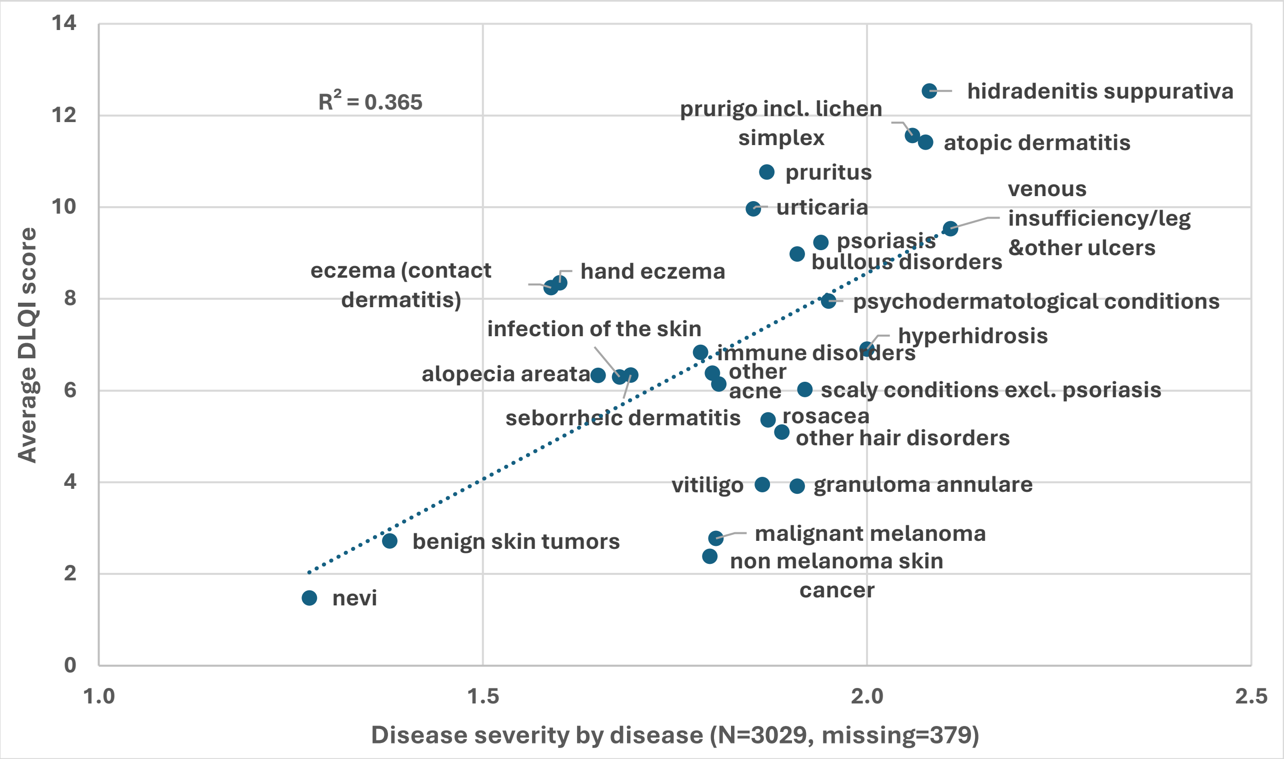

Table S1. Correlation matrix between DLQI items

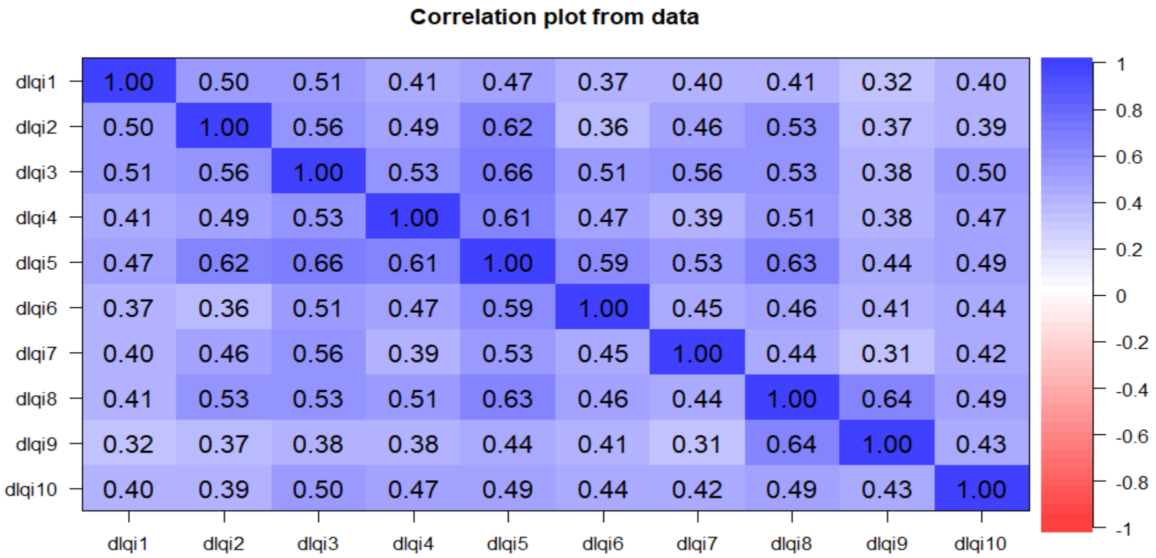
Correlation plot from data

	dlqi1	dlqi2	dlqi3	dlqi4	dlqi5	dlqi6	dlqi7	dlqi8	dlqi9	dlqi10
dlqi1	1.00	0.50	0.51	0.41	0.47	0.37	0.40	0.41	0.32	0.40
dlqi2	0.50	1.00	0.56	0.49	0.62	0.36	0.46	0.53	0.37	0.39
dlqi3	0.51	0.56	1.00	0.53	0.66	0.51	0.56	0.53	0.38	0.50
dlqi4	0.41	0.49	0.53	1.00	0.61	0.47	0.39	0.51	0.38	0.47
dlqi5	0.47	0.62	0.66	0.61	1.00	0.59	0.53	0.63	0.44	0.49
dlqi6	0.37	0.36	0.51	0.47	0.59	1.00	0.45	0.46	0.41	0.44
dlqi7	0.40	0.46	0.56	0.39	0.53	0.45	1.00	0.44	0.31	0.42
dlqi8	0.41	0.53	0.53	0.51	0.63	0.46	0.44	1.00	0.64	0.49
dlqi9	0.32	0.37	0.38	0.38	0.44	0.41	0.31	0.64	1.00	0.43
dlqi10	0.40	0.39	0.50	0.47	0.49	0.44	0.42	0.49	0.43	1.00

Table S2. Polychoric correlations of DLQI items

	dlqi1	dlqi2	dlqi3	dlqi4	dlqi5	dlqi6	dlqi7	dlqi8	dlqi9	dlq10
dlqi1	1									
dlqi2	0.58	1								
dlqi3	0.63	0.69	1							
dlqi4	0.51	0.6	0.66	1						
dlqi5	0.57	0.73	0.78	0.72	1					
dlqi6	0.51	0.5	0.65	0.62	0.74	1				
dlqi7	0.5	0.59	0.68	0.52	0.65	0.59	1			
dlqi8	0.52	0.66	0.66	0.64	0.75	0.62	0.57	1		
dlqi9	0.45	0.52	0.52	0.54	0.59	0.59	0.46	0.79	1	
dlqi10	0.52	0.51	0.62	0.6	0.62	0.59	0.54	0.62	0.59	1

Figure S6. Very Simple Structure plot using VSS promax rotation and minres factoring method

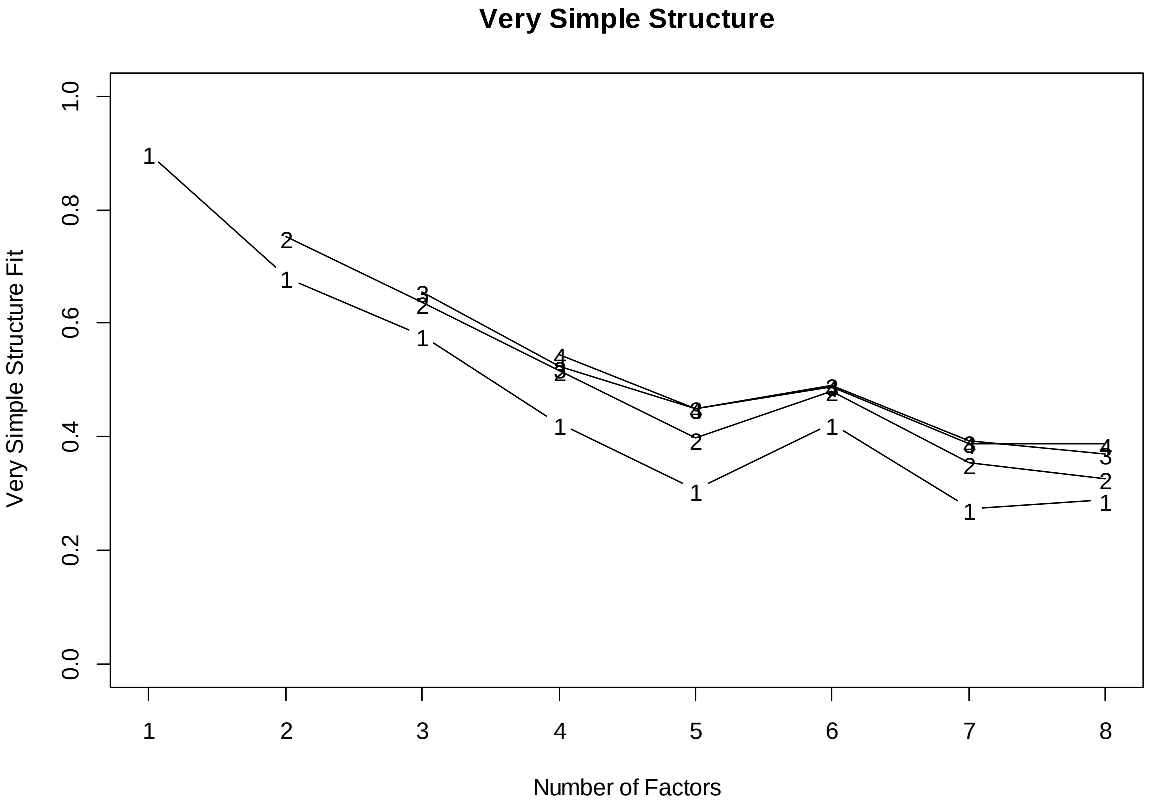

Table S3. Internal consistency reliability* of the DLQI

	Scale Mean if Item Deleted	Scale Variance if Item Deleted	Corrected Item-Total Correlation	Cronbach's Alpha if Item Deleted
dlqi1	5.57	37.233	0.577	0.893
dlqi2	5.66	36.035	0.657	0.888
dlqi3	6.11	36.236	0.737	0.883
dlqi4	6.03	36.640	0.651	0.888
dlqi5	6.01	35.455	0.783	0.879
dlqi6	6.23	37.805	0.617	0.890
dlqi7	6.06	36.861	0.599	0.892
dlqi8	6.18	37.259	0.710	0.885
dlqi9	6.31	38.893	0.547	0.895
dlqi10	6.19	38.191	0.609	0.891

* Cronbach's alpha

Table S4. Infit and outfit statistics (Rasch model)

	outfit	z.outfit	infit	z.infit
dlqi1	0.974	-1.042	1.080	3.287
dlqi2	0.812	-7.021	0.913	-3.645
dlqi3	0.557	-9.382	0.753	-9.151
dlqi4	0.795	-4.308	0.984	-0.551
dlqi5	0.499	-13.139	0.660	-13.913
dlqi6	0.700	-4.308	1.012	0.358
dlqi7	0.935	-1.18	1.138	4.334
dlqi8	0.575	-9.034	0.793	-7.261
dlqi9	0.874	-1.452	1.135	3.484
dlqi10	0.809	-3.506	1.018	0.605

Figure S7. Item response curves for the DLQI. Response levels are blue =0, orange=1, green=2, brown=3

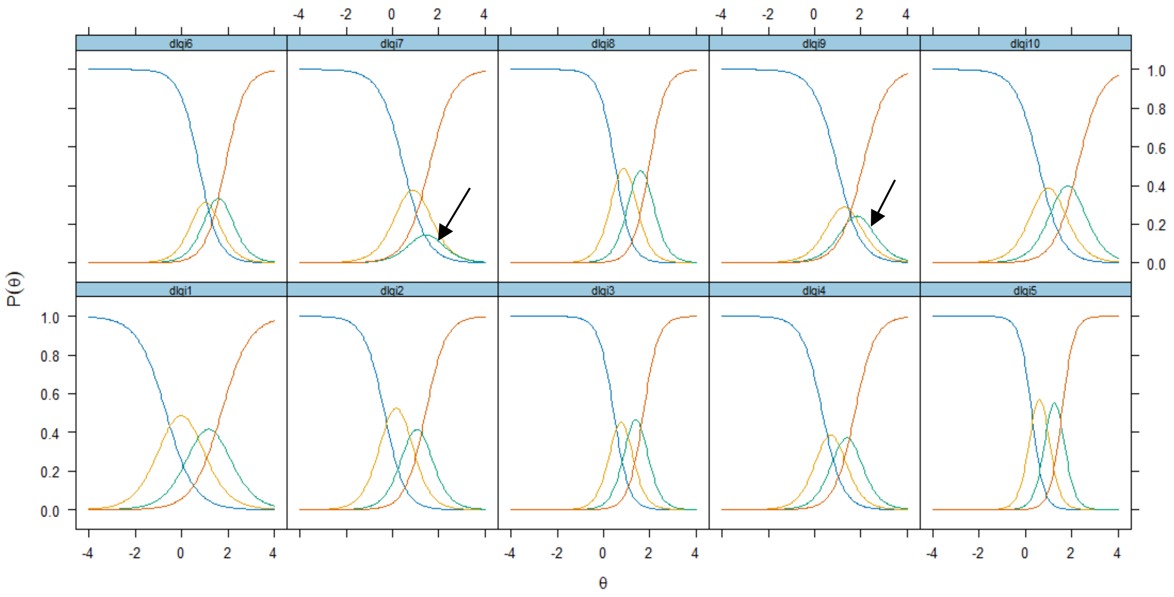

Figure S8. Test information (blue) and standard errors (red) for the DLQI response

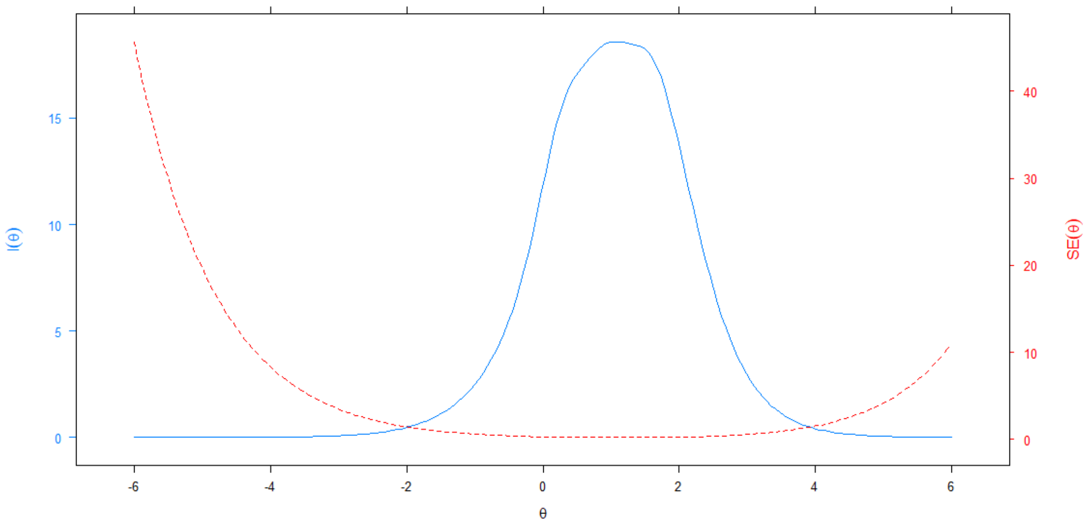

Figure S9. Observed versus expected values and empirical plots for DLQI items

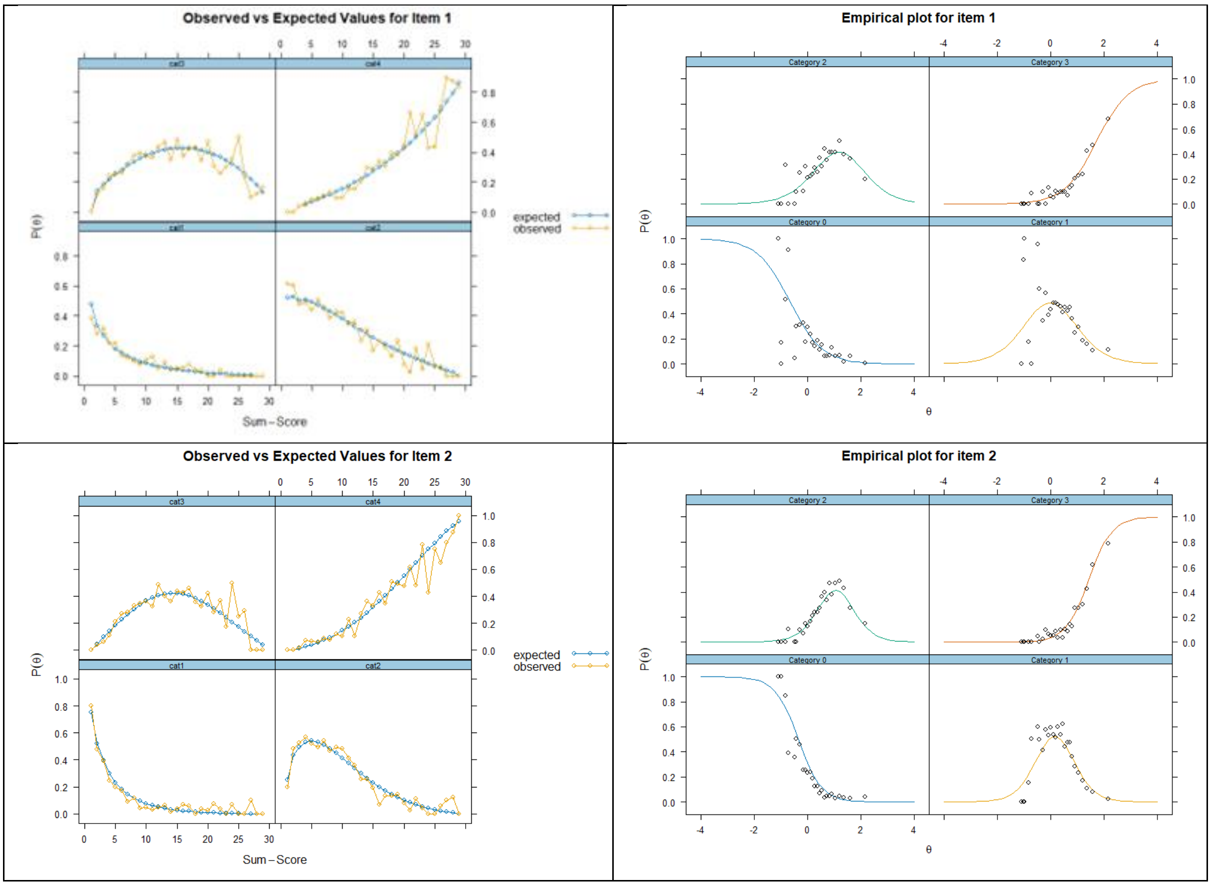

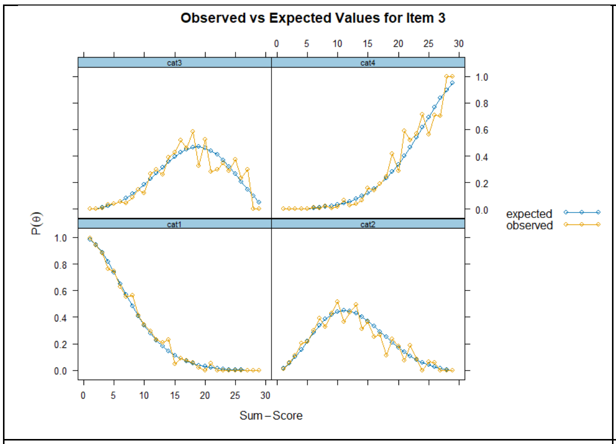
Observed vs Expected Values for Item 3
cat3
cat4
cat1
cat2
expected
observed
P(θ)
Sum – Score

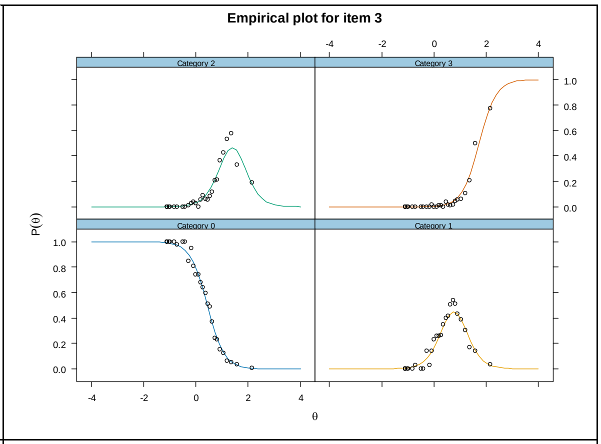
Empirical plot for item 3
Category 2
Category 3
Category 0
Category 1
P(θ)
θ

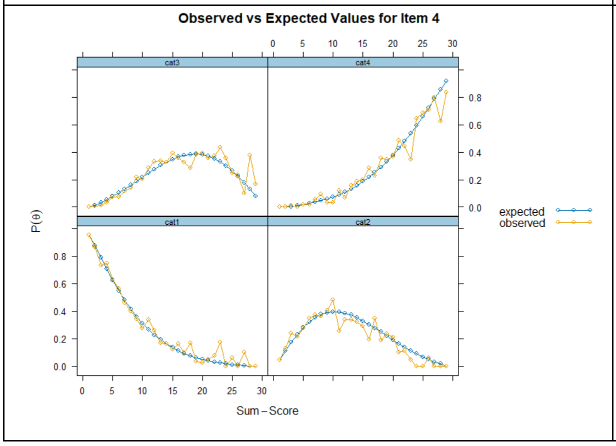
Observed vs Expected Values for Item 4
cat3
cat4
cat1
cat2
expected
observed
P(θ)
Sum – Score

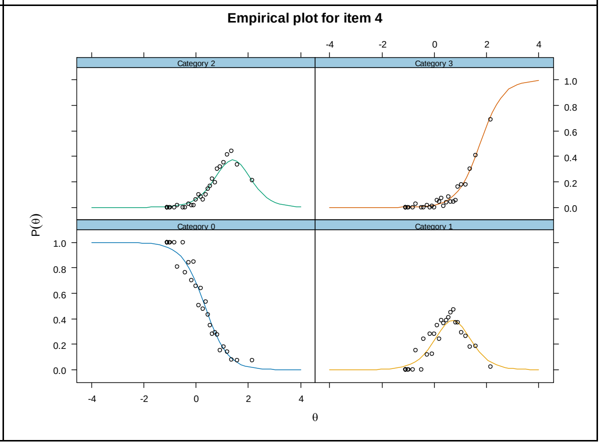
Empirical plot for item 4
Category 2
Category 3
Category 0
Category 1
P(θ)
θ

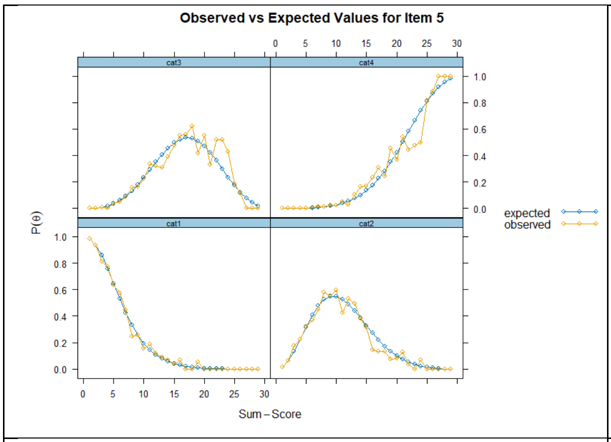
Observed vs Expected Values for Item 5
cat3
cat4
cat1
cat2
expected
observed
P(θ)
Sum – Score

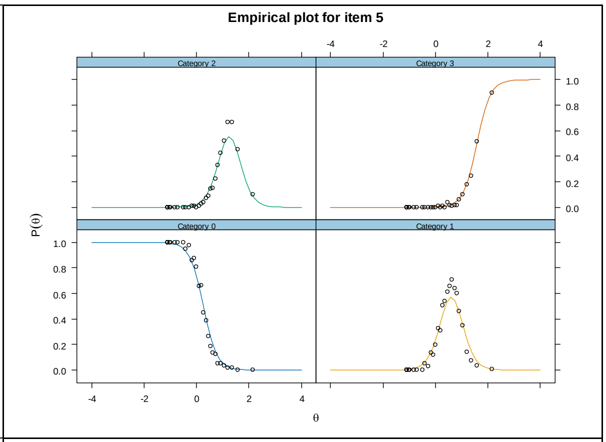
Empirical plot for item 5
Category 2
Category 3
Category 0
Category 1
P(θ)
θ

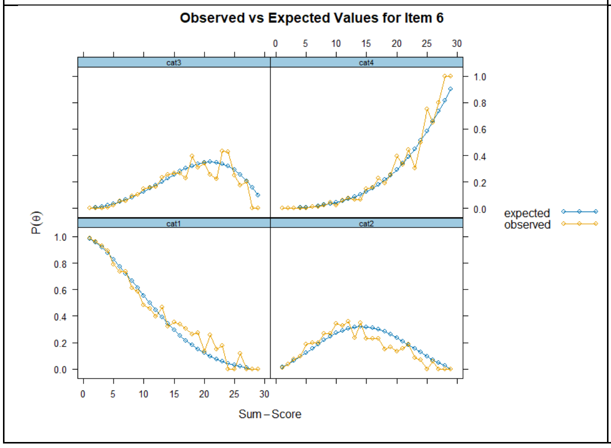
Observed vs Expected Values for Item 6
cat3
cat4
cat1
cat2
expected
observed
P(θ)
Sum – Score

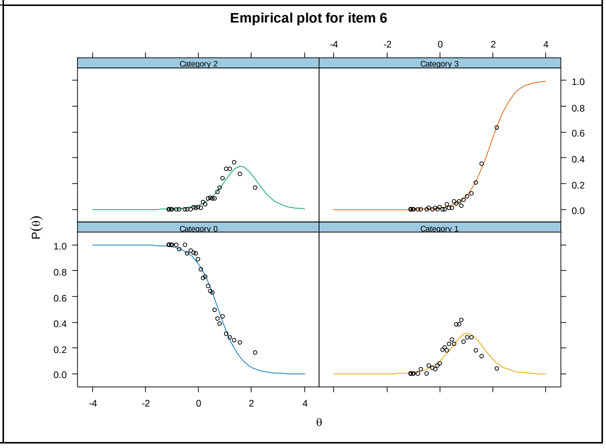
Empirical plot for item 6
Category 2
Category 3
Category 0
Category 1
P(θ)
θ

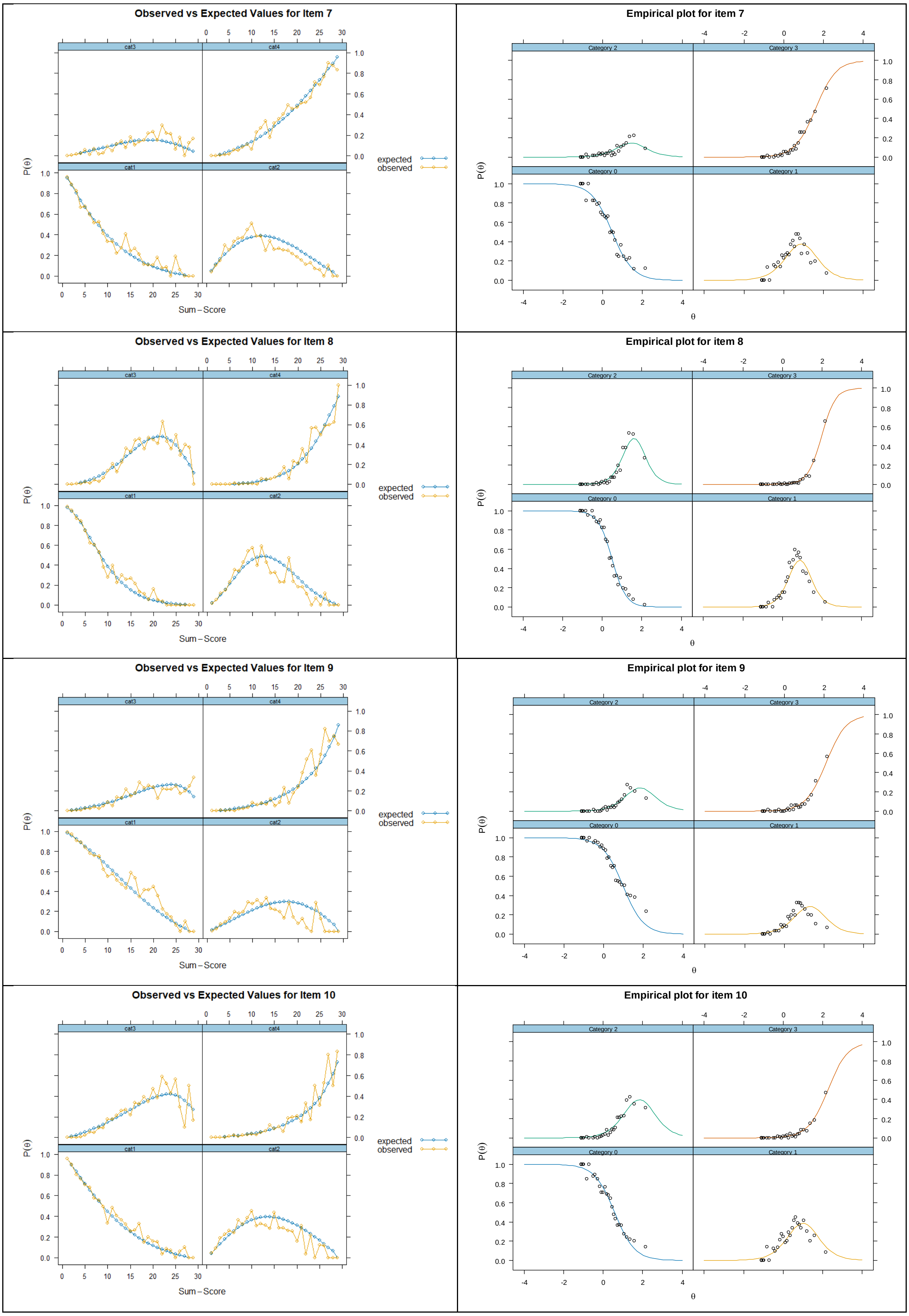
Observed vs Expected Values for Item 7
Empirical plot for item 7
Observed vs Expected Values for Item 8
Empirical plot for item 8
Observed vs Expected Values for Item 9
Empirical plot for item 9
Observed vs Expected Values for Item 10
Empirical plot for item 10
cat1
cat2
cat3
cat4
Category 0
Category 1
Category 2
Category 3
expected
observed
P(θ)
Sum – Score
θ

Table S5. Local dependence LD2

LD matrix (lower triangle) and standardized values

	dlqi1	dlqi2	dlqi3	dlqi4	dlqi5	dlqi6	dlqi7	dlqi8	dlqi9	dlqi10
dlqi1	NA	0.123	0.062	-0.063	-0.074	-0.071	-0.059	-0.079	-0.078	0.082
dlqi2	154.067	NA	-0.076	-0.09	0.094	-0.108	0.058	-0.089	-0.096	-0.109
dlqi3	39.754	58.341	NA	-0.069	-0.08	-0.098	0.09	-0.085	-0.1	-0.086
dlqi4	40.328	83.495	49.037	NA	0.081	-0.074	-0.074	-0.061	-0.083	0.076
dlqi5	55.92	89.771	65.334	66.745	NA	0.139	-0.07	-0.085	-0.114	-0.108
dlqi6	51.753	119.751	97.684	55.517	196.509	NA	-0.072	-0.07	-0.08	0.052
dlqi7	35.375	34.983	82.918	56.146	50.362	53.533	NA	-0.061	-0.076	-0.071
dlqi8	63.537	80.101	73.274	37.538	74.234	50.194	38.606	NA	0.152	-0.073
dlqi9	62.862	93.931	101.508	70.733	132.046	65.13	59.689	237.184	NA	0.067
dlqi10	68.272	121.397	75.366	59.644	119.63	27.583	51.896	55.141	46.065	NA

Local dependence exists when there is additional systematic covariance among the items. It can occur when pairs of items have highly similar content or between sequentially presented items in a test. No violation of local independence was seen as there were no residual correlations among the items after controlling for the dominant factor.

Table S6. Q3 statistics. All Q3 < 0.37

Q3	dlqi1	dlqi2	dlqi3	dlqi4	dlqi5	dlqi6	dlqi7	dlqi8	dlqi9	dlqi10
dlqi1	1	-0.007	0.045	-0.087	-0.195	-0.081	-0.036	-0.139	-0.076	-0.018
dlqi2	-0.007	1	-0.040	-0.105	-0.057	-0.263	-0.055	-0.062	-0.112	-0.180
dlqi3	0.045	-0.040	1	-0.146	-0.187	-0.139	0.065	-0.296	-0.255	-0.094
dlqi4	-0.087	-0.105	-0.146	1	-0.085	-0.049	-0.168	-0.123	-0.107	-0.007
dlqi5	-0.195	-0.057	-0.187	-0.085	1	-0.026	-0.137	-0.206	-0.237	-0.248
dlqi6	-0.081	-0.263	-0.139	-0.049	-0.026	1	-0.009	-0.180	-0.022	-0.031
dlqi7	-0.036	-0.055	0.065	-0.168	-0.137	-0.009	1	-0.170	-0.150	-0.032
dlqi8	-0.139	-0.062	-0.296	-0.123	-0.206	-0.180	-0.170	1	0.326	-0.069
dlqi9	-0.076	-0.112	-0.255	-0.107	-0.237	-0.022	-0.150	0.326	1	0.053
dlqi10	-0.018	-0.18	-0.094	-0.007	-0.248	-0.031	-0.032	-0.069	0.053	1

Figure S10. Known group validity analysis of DLQI total score by physician reported disease severity

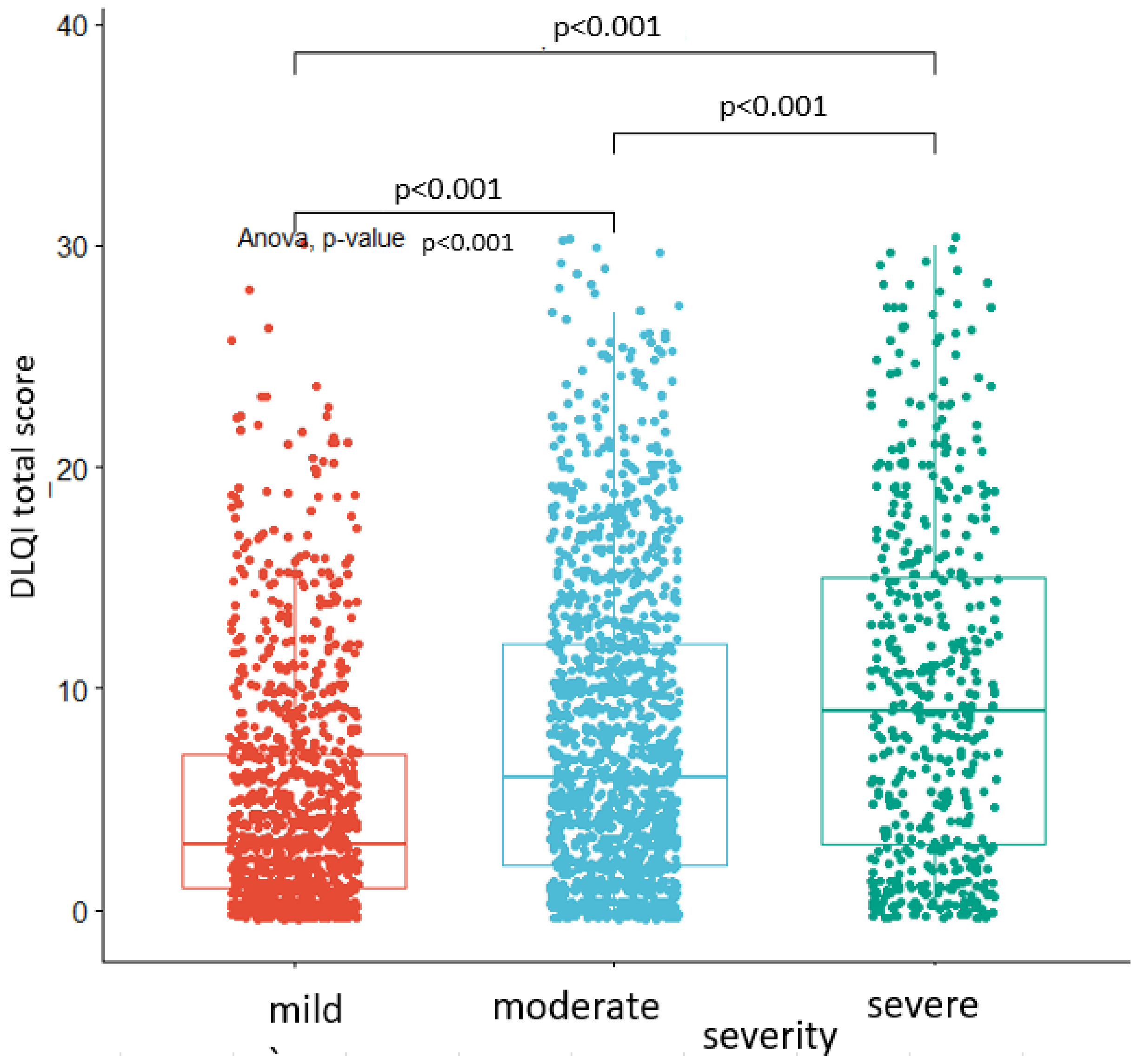

Supplement: Supplementary file 1 — Supplementary Material 1 [file 41687_2026_1025_MOESM1_ESM.pdf]
